# One-year pulmonary impairment after severe COVID-19: a prospective, multicenter follow-up study

**DOI:** 10.1186/s12931-022-01994-y

**Published:** 2022-03-21

**Authors:** Paola Faverio, Fabrizio Luppi, Paola Rebora, Gabriele D’Andrea, Anna Stainer, Sara Busnelli, Martina Catalano, Giuseppe Modafferi, Giovanni Franco, Anna Monzani, Stefania Galimberti, Paolo Scarpazza, Elisa Oggionni, Monia Betti, Tiberio Oggionni, Federica De Giacomi, Francesco Bini, Bruno Dino Bodini, Mara Parati, Luca Bilucaglia, Paolo Ceruti, Denise Modina, Sergio Harari, Antonella Caminati, Marcello Intotero, Pietro Sergio, Giuseppe Monzillo, Giovanni Leati, Andrea Borghesi, Maurizio Zompatori, Rocco Corso, Maria Grazia Valsecchi, Giacomo Bellani, Giuseppe Foti, Alberto Pesci

**Affiliations:** 1 Department of Medicine and Surgery, Università degli Studi di Milano Bicocca, Respiratory Unit, San Gerardo Hospital, ASST di Monza, via Pergolesi 33, 20900 Monza, Italy; 2 Bicocca Bioinformatics Biostatistics and Bioimaging B4 Center, University of Milano Bicocca, Monza, Italy; 3 Radiology Unit, Gerardo Hospital, ASST Monza, Monza, Italy; 4 Department of Biomedical Sciences, Humanitas University, Via Rita Levi Montalcini 4, Pieve Emanuele, 20072 Milan, Italy; 5 IRCCS Humanitas Research Hospital, Respiratory Unit, Via Manzoni 56, 20089 Rozzano, Milan Italy; 6 Division of Pulmonary Medicine, Civile Hospital, Vimercate, MB Italy; 7 Division of Pulmonary Medicine, Cremona Hospital, ASST Cremona, Cremona, Italy; 8 UOC Pulmonology, Department of Internal Medicine, Ospedale G. Salvini, ASST-Rhodense, Garbagnate Milanese, MI Italy; 9 Department of Pulmonology and Respiratory High-Dependency Unit, Ospedale Maggiore, Crema, Italy; 10 U.O. Pneumologia e Fisiopatologia Respiratoria-ASST Spedali Civili di Brescia, Brescia, Italy; 11 Department of Medical Sciences, San Giuseppe Hospital, MultiMedica IRCCS, Milan, Italy; 12 Department of Clinical Sciences and Community Health, Università degli Studi di Milano, Milan, Italy; 13 U.O. di Pneumologia e Terapia Semi-Intensiva Respiratoria, Servizio di Fisiopatologia Respiratoria ed Emodinamica Polmonare. Ospedale San Giuseppe-MultiMedica IRCCS, via San Vittore 12, 20123 Milan, MI Italy; 14 U.O.C. Radiologia, Civile Hospital, Vimercate, MB Italy; 15 U.O. Radiodiagnostica, Cremona Hospital, ASST Cremona, Cremona, Italy; 16 U.O.C. Radiodiagnostica, Ospedale G. Salvini, ASST-Rhodense, Garbagnate Milanese, MI Italy; 17 U.O.C. Radiologia, Ospedale Maggiore, Crema, Italy; 18 U.O. Radiologia, ASST Spedali Civili di Brescia, Brescia, Italy; 19 Dipartimento di Radiologia, Policlinico di Sant’Orsola, Alma Mater Studiorum-Università di Bologna, Bologna, Italy; 20 School of Medicine and Surgery, University of Milano Bicocca, Monza, Italy; 21 Department of Anesthesia and Intensive Care Medicine, ASST Monza, Monza, Italy

**Keywords:** COVID-19, Pneumonia, Pulmonary function test, Pulmonary fibrosis, High resolution computed tomography (HRCT)

## Abstract

**Background:**

Long-term pulmonary sequelae following hospitalization for SARS-CoV-2 pneumonia is largely unclear. The aim of this study was to identify and characterise pulmonary sequelae caused by SARS-CoV-2 pneumonia at 12-month from discharge.

**Methods:**

In this multicentre, prospective, observational study, patients hospitalised for SARS-CoV-2 pneumonia and without prior diagnosis of structural lung diseases were stratified by maximum ventilatory support (“oxygen only”, “continuous positive airway pressure (CPAP)” and “invasive mechanical ventilation (IMV)”) and followed up at 12 months from discharge. Pulmonary function tests and diffusion capacity for carbon monoxide (DLCO), 6 min walking test, high resolution CT (HRCT) scan, and modified Medical Research Council (mMRC) dyspnea scale were collected.

**Results:**

Out of 287 patients hospitalized with SARS-CoV-2 pneumonia and followed up at 1 year, DLCO impairment, mainly of mild entity and improved with respect to the 6-month follow-up, was observed more frequently in the “oxygen only” and “IMV” group (53% and 49% of patients, respectively), compared to 29% in the “CPAP” group. Abnormalities at chest HRCT were found in 46%, 65% and 80% of cases in the “oxygen only”, “CPAP” and “IMV” group, respectively. Non-fibrotic interstitial lung abnormalities, in particular reticulations and ground-glass attenuation, were the main finding, while honeycombing was found only in 1% of cases. Older patients and those requiring IMV were at higher risk of developing radiological pulmonary sequelae. Dyspnea evaluated through mMRC scale was reported by 35% of patients with no differences between groups, compared to 29% at 6-month follow-up.

**Conclusion:**

DLCO alteration and non-fibrotic interstitial lung abnormalities are common after 1 year from hospitalization due to SARS-CoV-2 pneumonia, particularly in older patients requiring higher ventilatory support. Studies with longer follow-ups are needed.

## Introduction

The Coronavirus disease 2019 (COVID-19) pandemic, caused by the severe acute respiratory syndrome coronavirus 2 (SARS-CoV-2) and initiated in December 2019, expanded dramatically throughout the world [1]. Pneumonia and acute respiratory distress syndrome (ARDS), frequent manifestations of COVID-19, may cause pulmonary sequelae including pulmonary fibrosis [2, 3]. Short-term pulmonary sequelae have been described in cohorts of patients followed up between 3 and 6 months after discharge and range from mild respiratory impairment, with moderately reduced DLCO in asymptomatic patients, to more significant restrictive ventilatory dysfunction in patients suffering persistent pulmonary symptoms, mainly exertional dyspnea [4, 5]. The severity of respiratory failure and the need of higher respiratory support (endotracheal intubation and invasive mechanical ventilation (IMV)) during pneumonia together with the extension of the radiological involvement were identified as factors associated to the development of pulmonary sequelae both functional and radiological [4–6].

A short-term follow-up may not be adequate to evaluate the long-term prognosis of respiratory impairment, therefore studies with longer follow-ups are warranted. Preliminary data from Chinese cohorts showed that up to 47% of patients showed residual abnormalities on pulmonary Computed Tomography (CT) scan performed at 1 year from the pneumonia, with ground glass attenuation and reticular abnormalities as the major radiologic patterns [7]. Furthermore, when comparing 6 to 12-month follow-up radiological exams, fibrotic interstitial lung abnormalities (ILA) and traction bronchiectasis remained stable, while non fibrotic ILA were completely or partially resolved [8]. The largest observational 1-year follow-up study available till now and performed in Wuhan, China, reported a prevalence of lung diffusion impairment up to 54% in critically ill patients and a significant burden of symptoms with 30% of patients still complaining of dyspnea [9]. However, large observational studies on long-term pulmonary sequeale in European cohorts are still missing.

Moreover, prior experience with the SARS due to SARS-CoV-1 reported the presence of pulmonary sequealae, although of mild entity, even years after the infection [10, 11].

This study aims to identify and characterize the pulmonary sequelae, in patients hospitalized for SARS-CoV-2 pneumonia, at 12 months follow-up after hospital discharge, and to evaluate their association with the maximum ventilatory support received during hospitalization.

## Materials and methods

### Study design and participants

In this multicenter, prospective, observational cohort study, we enrolled consecutive patients hospitalized for laboratory-confirmed SARS-CoV-2 pneumonia between March and June 2020 in 7 hospitals in Lombardy, a region of Northern Italy populated by about 10 million people: San Gerardo Hospital, Monza; G. Salvini Hospital, Garbagnate Milanese; San Giuseppe Hospital, Milan; Spedali Civili, Brescia; Ospedale Civile, Vimercate; Ospedale Maggiore, Crema; Ospedale Maggiore, Cremona. Patients were followed up at 6 and 12 months from discharge to evaluate the presence of pulmonary sequelae with clinical evaluation, complete pulmonary function tests (PFTs) including plethysmography and diffusion capacity for carbon monoxide (DLCO) with single-breath technique, 6-min walking test (6MWT), chest X-ray (only at 6-month follow-up) and high-resolution computed tomography (HRCT) (only at 12-month follow-up). Clinical evaluation included the collection of a dyspnea score (Modified Medical Research Council (mMRC) scale) and lung auscultation to detect the presence of pathologic lung sounds.

Patients were stratified according to the maximum oxygen/ventilatory support received during hospital stay: (1) oxygen therapy alone; (2) continuous positive airway pressure (CPAP); (3) invasive mechanical ventilation (IMV). CPAP and IMV were applied according to the position papers on the management of respiratory failure in patients with COVID-19 [12]. In our cohort, high-flow nasal cannula oxygen was only utilised in patients with moderate-to-severe acute respiratory failure as oxygen support in-between CPAP cycles. Patients in the “oxygen only” group presented a mild respiratory failure with a median [Q1–Q3] oxygen flow of 4 [2–6] l/min with nasal cannulae.

The study design planned two follow-up visits at 6 and 12 months from hospital discharge. Results from 6 months follow-up, as well as inclusion and exclusion criteria and study procedures, are summarised in the manuscript by Faverio et al*.* [4]. In the present paper we report results from the 12-month follow-up visit (visits were conducted in a time span ranging from 11 to 13 months after discharge with no differences between groups). This study received Ethics Committee approval (ASST Monza, 3389, May 21st 2020) and was registered on clinicaltrial.gov (ClinicalTrials.gov Identifier: NCT04435327). All patients provided written informed consent at the time of enrolment. The study is reported according to STROBE guidelines [13].

### High-resolution CT scan

HRCT scans were evaluated centrally by two senior radiologists (GDA and AP) of the referral center (San Gerardo Hospital, Monza) with over 20-year experience for the evaluation and quantization of interstitial lung diseases (ILDs), pulmonary fibrosis, emphysema and non-traction bronchiectasis. After independent evaluation, discussion and consensus resolved any possible disagreement. The following radiological scores were used: Oda et al. [14] and Ichikado et al. [15] for ILDs and pulmonary fibrosis and Fleischner Society classification system [16] for pulmonary emphysema. Isolated cystic lung alterations, pneumatoceles, large airways abnormalities and pulmonary artery enlargement were also evaluated. ILDs qualitative description according to the above cited scores included air-space consolidation, ground-glass opacities (GGO), honeycombing, reticular abnormalities (RA) and ground-glass attenuation with traction bronchiectasis.

For every type of radiological abnormality the localization based on lung lobes and the extension for every single lobe approximated in 10% intervals (10–20–30% etc.) was reported.

### Outcomes

The primary endpoint of the study was DLCO impairment (DLCO% < 80% of predicted) evaluated at 12 months from hospital discharge.

The secondary endpoints of the study were also assessed at 12 months from hospital discharge and were: (1) Vital Capacity (VC), Forced Vital Capacity (FVC), Tiffeneau Index (FEV1/FVC ratio), Forced Expiratory Volume in the 1st second (FEV1), Total Lung Capacity (TLC) and Residual Volume (RV) alterations; (2) dyspnea evaluated through mMRC scale; (3) radiological alterations on HRCT scan; and 4) variation from the expected of the normal distance walked on 6MWT.

### Statistical analysis

Baseline characteristics were described as median (I and III quartiles, Q1-Q3) and frequencies (percentage). Differences between the three strata identified by the maximum ventilatory support received during hospital stay were compared by Fisher’s exact test or Kruskal–Wallis rank sum test, as appropriate. PFT results within subjects were compared among follow-up visits by paired t-test, while dyspnea scale and categorical physical exams by McNeamer test.

In order to evaluate the association between the maximum ventilatory support and the presence of alterations at HRCT scans a logistic multivariable model was applied adjusting for predefined variables: age, gender, body mass index (BMI), cardiovascular diseases, diabetes, asthma, and treatment during hospital stay with systemic steroids or prophylactic heparin. A generalized estimating equations (GEE) approach has been undertaken in order to evaluate the association between maximum ventilatory support and DLCO impairment during follow-up (6 and 12 months), adjusting for the same predefined variables described above. Interactions were investigated and included in the model if statistically significant (p value < 0.05).

Results were reported as odds ratio (OR) with 95% confidence interval (CI). The analyses were performed in R (version 4.0.4) and SAS (v 9.4).

## Results

### Study population

**Fig. 1:**
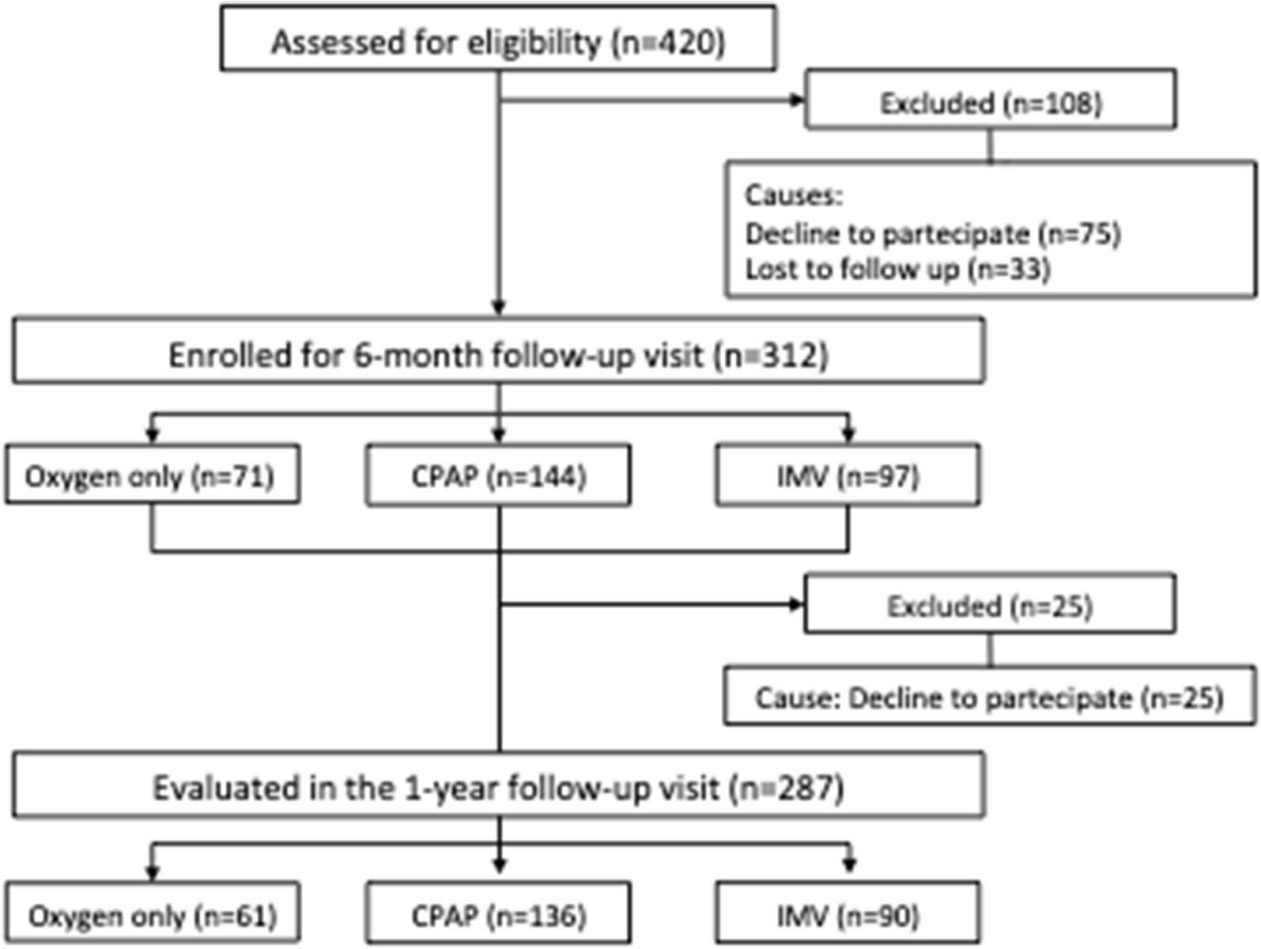
Study flow-chart. *CPAP* continuous positive airway pressure, *IMV* invasive mechanical ventilation

Out of the 420 consecutive hospitalized patients with SARS-CoV-2 pneumonia, 312 met inclusion and exclusion criteria, provided consent and were enrolled in the study. Out of these, 287 (92%) were followed up to 12-month (213, 74% men; median [Q1-Q3] age 60.7 [53.4–68.8] years) and were stratified as follows: 61 patients in the “oxygen alone” group, 136 patients in the “CPAP” group and 90 patients in the “IMV” group, Fig. 1. Among the 25 patients that declined to participate to the second follow-up, none died between the 6-month and the 12-month visit. However, 5 patients (3 in the oxygen only group and 2 in the CPAP group) were re-hospitalized between the 6 and 12-month follow-up visit. Causes of re-hospitalization were: perianal abscess, acute renal failure secondary to diarrhea, inguinal hernioplasty, intestinal ischemia, acute cryptogenic cerebral ischemia.

The baseline clinical features of the study population stratified by maximum oxygen/ventilatory support are shown in Table 1. The majority of patients were never smokers (163, 68%) with only one or absence of comorbidities (222, 78%). The most frequently encountered comorbidities were obesity (35%), hypertension (29%), cardiovascular diseases (23%) and diabetes (14%). In regards to treatments received during hospitalization for COVID-19, patients in the “oxygen alone” group received significantly less specific treatments compared to the other groups. Pulmonary thromboembolism and deep vein thrombosis, two possible complications of COVID-19, were reported in 13 (4.5%) and 3 (1%) patients, respectively, with no differences between groups.

**Table 1:** Demographics and clinical characteristics of study cohort at baseline

	Oxygen only (N = 61)	CPAP (N = 136)	IMV (N = 90)	p
Age (years), median [Q1–Q3]	60.7 [53.7, 71.4]	60.7 [53.0, 67.5]	60.3 [54.4, 67.0]	0.46
Male gender, N (%)	33 (54)	106 (78)	74 (82)	< 0.001
BMI (kg/m^2^), median [Q1–Q3]	27.4 [24.5, 31.5]	28.7 [26.6, 31.3]	28.3 [26.3, 31.4]	0.15
Smoking History^a^, N (%)				0.09
No	40 (85)	76 (64)	47 (64)	
Active-prior	7 (11)	43 (32)	26 (29)	
Comorbidities				
Cardiovascular diseases, N (%)	10 (16)	31 (23)	24 (27)	0.32
Hypertension, N (%)	19 (31)	39 (29)	24 (27)	0.82
Cerebrovascular diseases, N (%)	1 (2)	3 (2)	1 (1)	1.00
Asthma, N (%)	8 (13)	4 (3)	4 (4)	0.02
OSAS, N (%)	2 (3)	3 (2)	1 (1)	0.76
Chronic kidney diseases, N (%)	4 (7)	2 (1)	3 (3)	0.12
Liver diseases, N (%)	1 (2)	3 (2)	0 (0)	0.43
Diabetes, N (%)	10 (16)	18 (13)	13 (14)	0.84
Prior cancer, N (%)	5 (8)	2 (1)	5 (6)	0.05
No. of comorbidities, N (%)				-
0	20 (33)	64 (47)	36 (40)	
1	26 (43)	42 (31)	34 (38)	
2	9 (15)	23 (17)	16 (18)	
≥ 3	6 (10)	7 (5)	4 (4)	
Treatments associated with COVID-19				
Systemic steroid^b^, N (%)	15 (30)	62 (56)	44 (59)	0.002
Prophylactic heparin^b^, N (%)	15 (30)	53 (48)	41 (55)	0.02
Tocilizumab^b^, N (%)	3 (6)	17 (15)	17 (23)	0.04
Remdesivir^c^, N (%)	1 (2)	2 (2)	11 (15)	0.001
Mucolytics^c^, N (%)	10 (20)	32 (29)	35 (47)	0.004
Hyperimmune Plasma^c^, N (%)	0 (0)	1 (1)	1 (1)	1.000
Lopinavir/ritonavir^c^, N (%)	19 (38)	73 (66)	36 (49)	0.002
Hydroxychlorokine^d^, N (%)	39 (78)	95 (87)	58 (79)	0.23

*BMI* body mass index, *CPAP* continuous positive airway pressure, *IMV* invasive mechanical ventilation, *Q1–Q3* first-third quartile, *OSAS* obstructive sleep apnea syndrome

^a^48 missing

^b^52 missing

^c^53 missing

^d^55 missing

The median (Q1-Q3) hospital length of stay for each study groups was 10 (6–14), 17 (16–22) and 33 (26–43) days for “oxygen only”, CPAP and IMV, respectively. In the IMV group, median (Q1–Q3) intensive care unit length of stay was 13 [10–15] days and median (Q1–Q3) duration of IMV was 11 [8–12] days.

### Evaluation of pulmonary sequelae

**Fig. 2:**
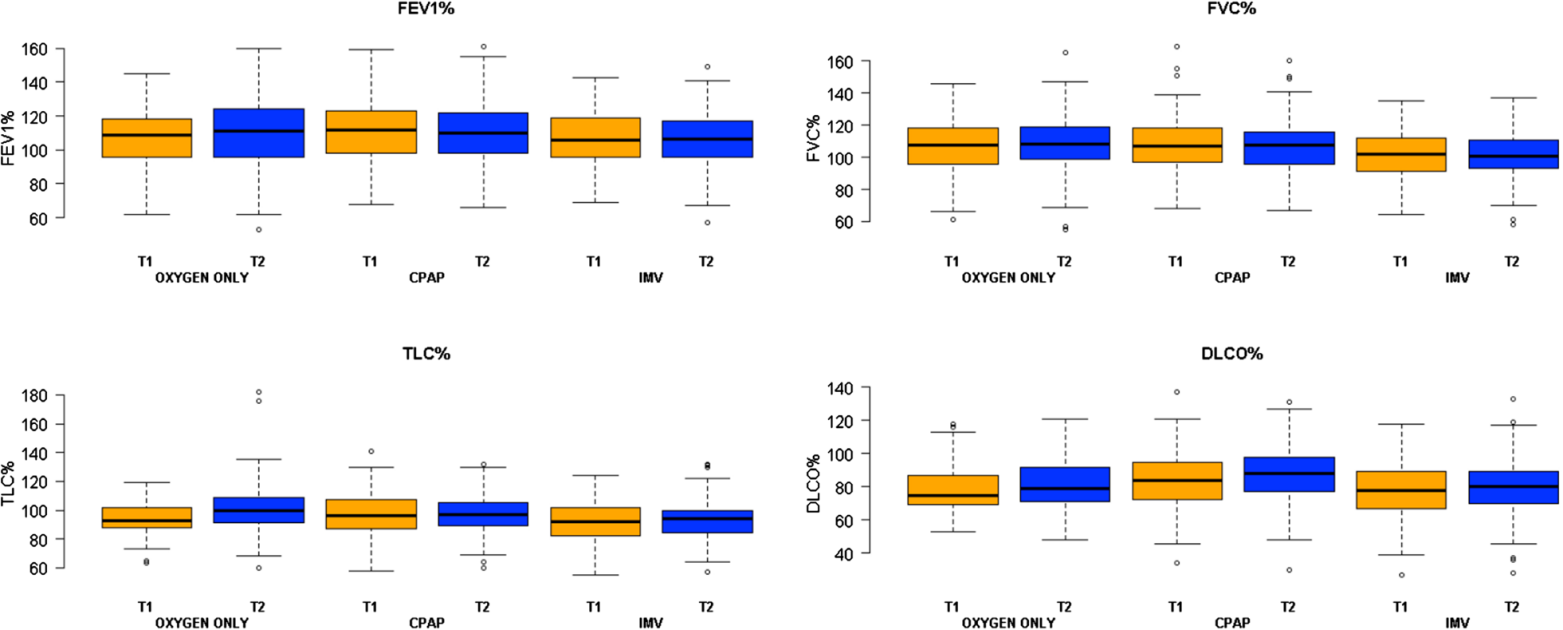
Comparison of the main pulmonary function tests between 6- and 12-month follow-up visit. *FEV1* forced expiratory volume in the 1st second, *FVC* forced vital capacity, *TLC* total lung capacity, *DLCO* diffusion capacity for carbon monoxide, *CPAP* continuous positive airway pressure, *IMV* invasive mechanical ventilation

In regards to the presence of DLCO impairment, we found similar results to the 6-month follow-up with the highest prevalence of DLCO alteration in the “oxygen alone” (n = 31, 53%) and “IMV” group (n = 44, 49%) and the lowest in the “CPAP” group (n = 39, 29%), Table 2. DLCO improved between 6- and 12-month follow-up in all 3 groups, although the improvement was statistically significant only for the CPAP and IMV group (mean difference between DLCO% at 12 and 6 months: 1.6%, 95% CI: − 2.0; 5.3, in the oxygen only group, 2.4%, 95% CI: 0.5; 4.2% in the CPAP group and 2.7%, 95% CI: 0.3; 5.0 in the IMV group), Fig. 2.

**Table 2:** Pulmonary function tests and dyspnea scale at 1 year from hospital discharge

	Oxygen only (N = 61)	CPAP (N = 136)	IMV (N = 90)	p
	Median [Q1–Q3]	Median [Q1–Q3]	Median [Q1–Q3]	
FEV1 (L)^a^	2.9 [2.4, 3.7]	3.4 [2.7, 3.9]	3.2 [2.7, 3.7]	
FEV1%	111.0 [96.0, 123.5]	110.0 [98.0, 121.8]	106.5 [96.2, 117.0]	0.24
FVC (L)^b^	3.4 [3.0, 4.5]	4.2 [3.3, 4.8]	3.9 [3.3, 4.6]	
FVC%	108.0 [99.0, 119.0]	107.5 [96.0, 116.0]	101.0 [93.0, 111.0]	0.02
TI^b^	80.0 [77.0, 84.0]	82.0 [79.0, 85.8]	82.0 [80.0, 85.0]	
TLC (L)^c^	5.8 [4.6, 7.1]	6.3 [5.3, 6.9]	5.9 [4.9, 6.7]	
TLC%	100.0 [91.0, 109.0]	97.0 [89.0, 105.2]	94.0 [84.0, 100.0]	0.02
DLCO (mmoL/min/kPa)^d^	6.4 [5.6, 7.6]	7.5 [6.3, 9.1]	6.9 [5.9, 8.5]	
DLCO%^c^	79.0 [71.2, 91.8]	88.0 [77.0, 98.0]	80.0 [70.2, 89.0]	0.006

	N (%)	N (%)	N (%)	
Pulmonary function test values as categorical variables				
DLCO impairment (%)	31 (53)	39 (29)	44 (49)	0.001
Mild defect (60–79%)	28 (48)	26 (20)	35 (39)	
Moderate defect (40–59%)	3 (5)	12 (9)	6 (7)	
Severe defect (< 40%)	0 (0)	1 (1)	3 (3)	
FVC impairment (%)	4 (7)	7 (5)	9 (10)	0.37
Mild defect (70–79%)	1 (2)	6 (4)	7 (8)	
Moderate defect (60–69%)	1 (2)	1 (1)	1 (1)	
Moderate-to-severe defect (50–59%)	2 (3)	0 (0)	1 (1)	
Severe defect (≤ 49%)	0 (0)	0 (0)	0 (0)	
TI < 0.7	5 (8)	6 (4)	0 (0)	0.02
TLC impairment (%)	6 (10)	11 (8)	16 (18)	0.09
Mild defect (70–79%)	4 (7)	8 (6)	11 (12)	
Moderate defect (60–69%)	2 (3)	3 (2)	4 (4)	
Moderate-to-severe defect (50–59%)	0 (0)	0 (0)	1 (1)	
Severe defect (≤ 49%)	0 (0)	0 (0)	0 (0)	
Six-minute walking test				
Meters, median [Q1–Q3]^e^	470 [400, 513]	460 [410, 520]	475 [400, 525]	0.60
Distance lower than expected^e^	15 (25)	26 (20)	17 (19)	0.64
Dyspnea scale				
mMRC SCALE^f^				0.81
0	35 (57)	83 (61)	53 (60)	
1	18 (30)	32 (24)	27 (30)	
2	8 (13)	17 (12)	9 (10)	
3	0 (0)	3 (2)	0 (0)	
4	0 (0)	1 (1)	0 (0)	

*CPAP* continuous positive airway pressure, *DLCO* diffusion capacity for carbon monoxide, *FEV1* forced expiratory volume in the 1st second, *FVC* forced vital capacity, *IMV* invasive mechanical ventilation, *Q1* first quartile, *Q3* third quartile, *RV* residual volume, *TI* tiffeneau Index (FEV1/FVC ratio), *TLC* total lung capacity, *VC* vital capacity. The lower limits of normal for distance walked in healthy men and women were calculated according to the equation created by Enright et al. [17]

^a^3 missing

^b^2 missing

^c^6 missing

^d^8 missing

^e^5 missing

^f^1 missing

When considering FVC and TLC as continuous variables, patients in the “IMV” group showed lower values compared to “CPAP” and “oxygen only” group, Table 2. However, only a minority of patients (20, 7%), with no differences between groups, showed a restrictive pattern, defined as having a normal FEV1/FVC and a FVC < 80% predicted [18]. An obstructive pattern (defined as Tiffeneau Index < 0.7 with a concomitant reduction of FEV1 < 80%) was observed only in 11 (3.8%) patients, one was active and three prior smokers and one had asthma as comorbidity. We observed no differences between 6- and 12-month follow-up in FEV1 and FVC, while TLC improved significantly in the “oxygen only” group (mean difference between values at 12- and 6 months 2.6%, 95% CI: 1.2; 4.0%), Fig. 2.

Median distance walked at 6MWT ranged between 460 and 475 m, with no differences between groups, Table 2. However, 58 (20%) patients showed a distance walked lower than expected, again without differences between groups. No patients showed oxygen desaturation or required oxygen supplementation during the test.

**Fig. 3:**
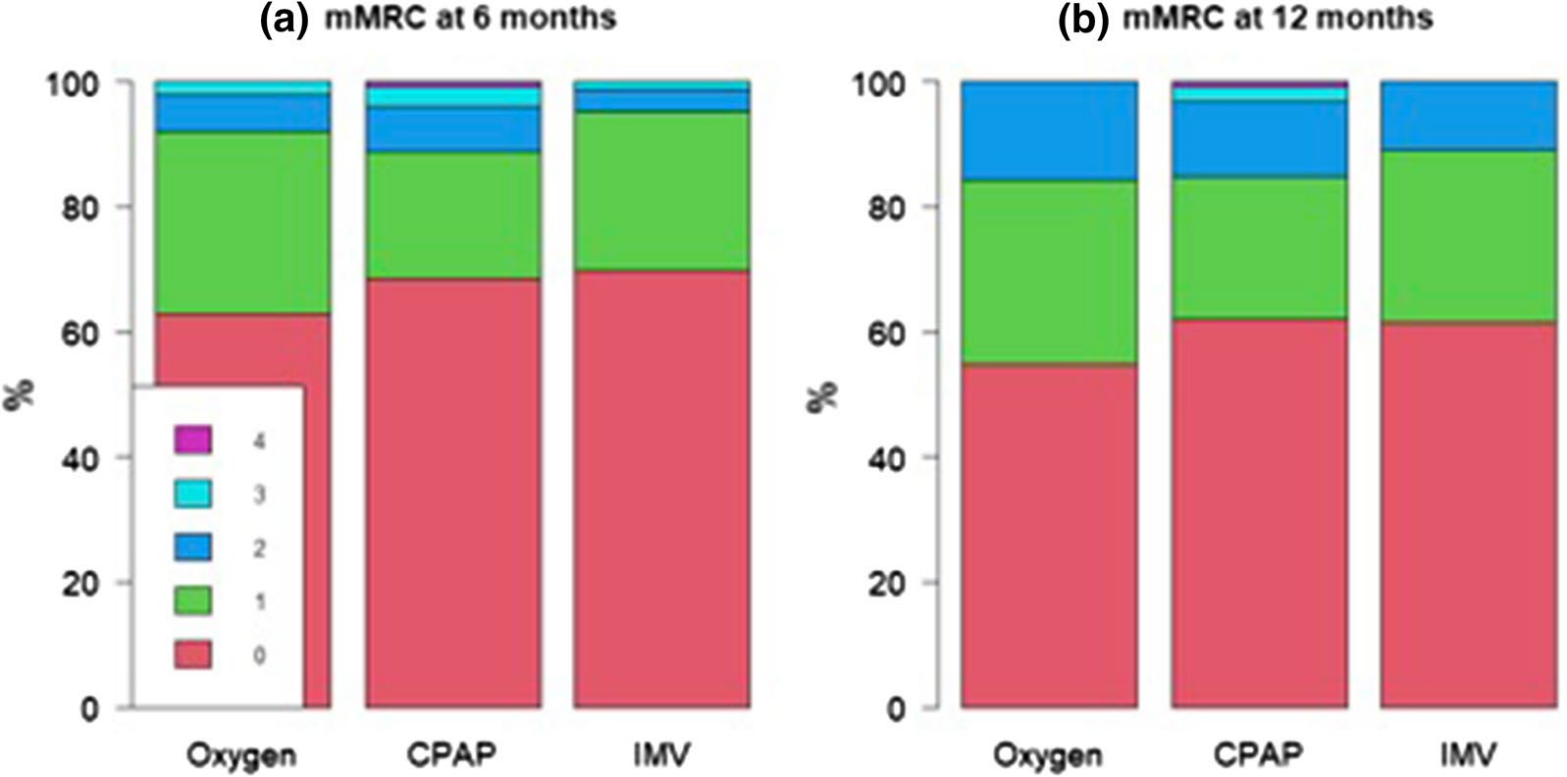
Comparison of mMRC dyspnea scale (grade 0 to 4 in the Figure Panel) between 6- and 12-month follow-up visit (n = 258 patients with no missing in *mMRC* at any follow-up visit). *mMRC* modified Medical Research Council, *CPAP* continuous positive airway pressure, *IMV* invasive mechanical ventilation

Characterizing the degree of dyspnea reported by patients through the mMRC scale, 115 (40%) still showed some degree of breathlessness, mainly mMRC grade 1 in 77 cases (“Dyspnea when hurrying or walking up a slight hill”) and mMRC grade 2 in 34 cases (“Walks slower than people of the same age because of dyspnea or has to stop for breath when walking at own pace”), with no differences between groups, Table 2. We observed an increase in the reported breathlessness, particularly mMRC grade 1 and 2, between 6- and 12-month visit: 19% vs 23% reporting any grade of dyspnea out of the 51 patients of the “oxygen only” group with no missing in mMRC at any follow-up visit, 31% vs 38% in the CPAP group and 30% vs 39% in the IMV group, with no statistical significance, Fig. 3. None of the patients underwent a rehabilitation program between 6 and 12 months follow-up.

After adjusting for demographics, comorbidities and treatments during hospital stay, Table 3, the IMV group showed higher odds of DLCO impairment during follow-up with respect to the “oxygen only” group, although the difference was not significant (OR = 1.44, 95% CI: 0.71; 2.93, p = 0.32). Interestingly, in subjects treated with prophylactic heparin the odds of DLCO alteration was reduced with a trend toward statistical significance (OR = 0.62, 95% CI: 0.38; 1.02, p = 0.06). DLCO impairment had a decreasing trend between the two visits but not statistically significant (OR = 0.83, 95% CI: 0.63; 1.09, p = 0.17).

**Table 3:** Multivariable model results on DLCO impairment during follow-up

DLCO impairment at 6 and 12 months	OR	95% CI	p
CPAP vs oxygen alone	0.73	0.39–1.35–	0.315
IMV vs oxygen alone	1.44	0.71–2.93	0.316
12-month vs 6-month visit	0.83	0.63–1.09	0.17
Age (per year) in males	0.99	0.96–1.03	0.534
Age (per year) in females	1.04	1.01–1.07	
BMI (per kg/m^2^)	0.94	0.88–1.01	0.078
Cardiovascular diseases (yes vs no)	0.76	0.41–1.38	0.360
Diabetes (yes vs no)	1.82	0.82–4.04	0.139
Asthma (yes vs no)	3.33	1.19–9.32	0.022
Systemic steroid (yes vs no)	1.50	0.90–2.50	0.125
Prophylactic heparin (yes vs no)	0.62	0.38–1.02	0.060

*BMI* body mass index, *CI* confidence intervals, *CPAP* continuous positive airway pressure, *DLCO* diffusion capacity for carbon monoxide, *IMV *invasive mechanical ventilation, *OR* odds ratio

### Evaluation of HRCT

Out of 287 patients who performed the 1-year visit, 17 (6%) refused to perform the HRCT scan. Of the remaining 270 patients, those receiving IMV had a higher percentage of pathological HRCT scans (n = 68, 80% vs n = 84, 65% in the CPAP group and n = 26, 46% in the “oxygen only” group, p < 0.001). This was confirmed by a multivariable adjusted model: in particular, in patients treated with IMV with respect to patients on oxygen alone (OR = 8.34, 95% CI: 2.97; 23.44, p < 0.001) and in those treated with “CPAP” compared to oxygen alone (OR = 2.78, 95% CI: 1.16; 6.66, p = 0.02). The odds of presenting radiological abnormalities on HRCT were also higher in older patients (OR = 1.07, 95% CI: 1.03; 1.1, p < 0.001).

**Fig. 4:**
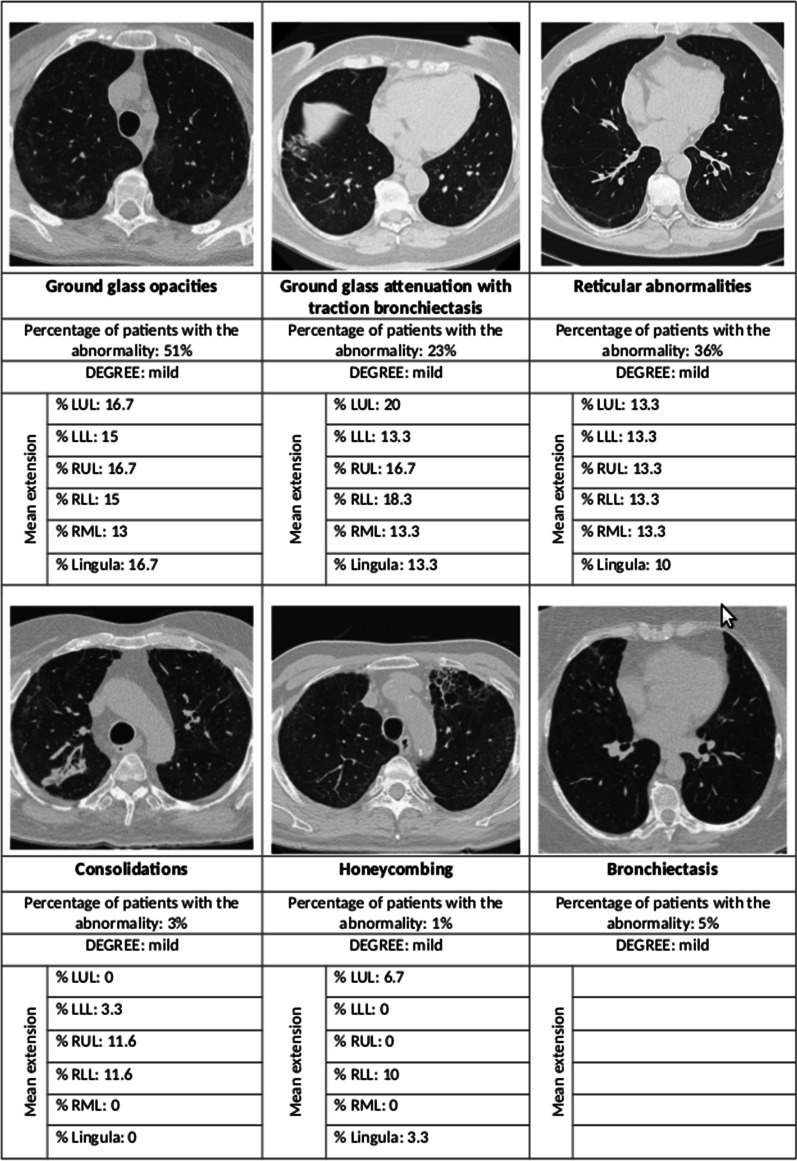
Summary of the main radiological abnormalities and their extension according to the lung lobe involved. *LUL* left upper lobe, *LLL* left lower lobe, *RUL* right upper lobe, *RLL* right lower lobe, *RML* right middle lobe

The majority of cases showed interstitial lung involvement with GGO (139 cases, 51%), followed by RA (98, 36%), consolidations (8, 3%) and honeycombing (3, 1%), Table 4 and Fig. 4. In 44% (61/139) of cases GGO was associated with traction bronchiectasis or bronchiolectasis. Crazy-paving and organizing pneumonia pattern (perilobular pattern, reversed halo sign or halo sign) were not observed. Both GGO and RA were more common in the IMV group. The mean anatomical extension of radiological alterations per lobe was 17% in Right Upper Lobe, Right Lower Lobe and lingula, 15% in Left Upper Lobe and Right Middle Lobe, and 13% in Left Lower Lobe.

**Table 4:** Chest HRCT scan characteristics of study cohort (17 patients did not perform HRCT and are not considered here)

	Oxygen only (N = 56)	CPAP (N = 129)	IMV (N = 85)	p
	N(%)	N(%)	N(%)	
Abnormal chest HRCT, N (%)	26 (46)	84 (65)	68 (80)	< 0.001
Abnormalities				
Air-space consolidation	2 (4)	4 (3)	2 (2)	0.90
Ground-glass attenuation	17 (30)	62 (48)	60 (71)	< 0.001
GGO with traction bronchiectasis	9	28	24	
Reticular abnormalities	15 (27)	41 (32)	42 (49)	0.01
Honeycombing	0 (0)	2 (2)	1 (1)	1.00
Emphysema	1 (2)	18 (14)	11 (13)	0.02
Centrilobular emphysema	1 (100)	14 (65)	7 (64)	1.00
Panlobular emphysema	0 (0)	1 (10)	0 (0)	
Paraseptal emphysema	0 (0)	3 (25)	7 (70)	
Bronchiectasis^a^	2 (4)	3 (2)	9 (11)	0.03
Medium^b^ bronchial enlargement	0 (0)	1 (33)	7 (78)	
Moderate^c^ bronchial enlargement	2 (100)	1 (33)	1 (11)	
Severe^d^ bronchial enlargement	0 (0)	1 (33)	1 (11)	
Organizing pneumonia	0	0	0	–
Lung lobes involved				
Left upper lobe	14 (25)	46 (36)	56 (66)	–
Right upper lobe	15 (27)	55 (43)	56 (66)	–
Right middle lobe	6 (11)	24 (19)	39 (46)	–
Lingula	4 (7)	19 (15)	36 (42)	–
Left lower lobe	16 (29)	53 (41)	39 (46)	–
Right lower lobe	20 (36)	60 (47)	47 (55)	–

*HRCT* high-resolution computed tomography, *GGO* ground glass opacities

^a^Exclusion of traction bronchiectasis

^b^Luminal diameter slightly larger than the adjacent vessel

^c^Bronchial diameter between 2 and 3 times the diameter of the adjacent vessels

^d^Bronchus is more than 3 times the diameter of the adjacent vessel

In patients presenting RA, the most common radiological features were subpleural curvilinear lines, with 1–3 mm thickness, lying less than 1 cm from and parallel to the pleural surface, that were observed in 71% of patients with RA, Fig. 4.

Emphysema was detected in a minority of patients (30, 11%) and was more frequent in those who underwent CPAP and IMV compared to the “oxygen only” group. Out of the 30 patients with emphysema 16 were prior smokers, 6 active smokers and 8 never smokers, none was asthmatic. Centrilobular emphysema was the most common (22 cases). Non-traction bronchiectasis were also observed in a minority of patients (14, 5%), mainly in the IMV group, and were of mild entity in the majority of cases.

Isolated cystic lung alterations and pneumatoceles as well as large airway diseases, including tracheomalacia, were not observed. Pulmonary artery enlargement was observed in 4 cases (3 in the CPAP group and 1 in the IMV group) but none of these patients had a diagnosis of pulmonary thromboembolism during hospitalization for COVID-19.

We also evaluated the correlation between DLCO impairment and the presence of HRCT abnormalities. Among the 158 patients with no DLCO impairment, 92 (58%) presented an Abnormal Chest HRCT, while among 106 patients with DLCO impairment, 82 (77%) presented an Abnormal Chest HRCT (chi-square p-value = 0.0013). Among the 158 patients with no DLCO impairment, 71 (45%) presented ground-glass opacities at HRCT scan, while among 106 patients with DLCO impairment, 65 (61%) presented ground-glass opacities at HRCT scan (chi-square p-value = 0.009).Among the 158 patients with no DLCO impairment, 45 (28%) presented reticular abnormalities at HRCT scan, while among 106 patients with DLCO impairment, 52 (49%) presented reticular abnormalities at HRCT scan (Chi-square p-value = 0.0007).

## Discussion

In our cohort of 287 patients at 12-month follow-up from hospitalization due to SARS-CoV-2 pneumonia fibrotic sequelae at HRCT scans were found in a strict minority of patients (3, 1% of the cohort). Mild non-fibrotic radiological abnormalities were observed in the majority of cases (66% of the entire cohort) with interstitial lung involvement, particularly GGO and RA, as subpleural curvilinear lines, as the main radiologic pattern. However, the anatomical extension of these abnormalities was limited, with a mean lobar involvement that ranges between 13 and 17% of each entire single lobe. Similar results were observed in a Chinese cohort of 41 patients where GGO and RA were the most common HRCT finding, although only 47% of the cohort showed residual radiological aberration [7]. Only a narrow minority of patients developed fibrotic sequelae (honeycombing was observed in 1% of cases) and irreverisible abormalities such as bronchiectasis, however we cannot predict the evolution of the more common non-fibrotic sequelae (mainly GGO and RA) and studies with longer follow-ups are required. Furthermore, it is difficult to identify whether the lung damage is entirely due to the viral action or is at least partially secondary to baro- and volotrauma during IMV. In fact, while we did not observe cystic alterations and pneumatoceles in our cohort, fibrotic sequelae might also be favored by barotrauma [19, 20].

In our cohort older patients with more severe pneumonia were at higher risk of developing radiological sequelae, which nicely fit with the results from a Chinese cohort by Chen et al. [7].

Almost 40% of patients showed DLCO impairment of mild entity in the majority of cases, and an even smaller percentage showed a restrictive pattern (between 7 and 11.5% according to the definition used). DLCO impairment was more common in patients in the “oxygen only” and IMV group. However, the number of patients in the “oxygen only” group was limited and the proportion of patients lost to follow-up was slightly higher compared to the other groups, which may have led to the selection of the most severe cases. Also interesting to note for the primary end-point, the DLCO values improved between the 6- and 12-month evaluation. Despite the mild entity of the functional sequelae, a consistent proportion of patients at 1-year from SARS-CoV-2 pneumonia still report exertional dyspnea (35%) with a worsening trend compared to the 6-month visit. Similar results were reported by the largest 1-year follow-up cohort described to date (1276 patients): Huang et al*.* observed a general improvement in functional and radiological lung sequelae between 6- and 12-month follow-up visits, however the number of patients with exertional dyspnea slightly increased between the two time-points reaching 30% of the entire cohort [9]. We did not collect other non-respiratory symptoms in our patients, however Huang et al*.* reported a slight increase also in anxiety and depression between 6 and 12 months after hospitalization for pneumonia. Furthermore, a recent study on the main symptoms reported by patients 1-year after COVID-19 hospitalization found that fatigue, anxiety and myalgia were among the most common [21]. All these debilitating symptoms were recently included in the definition of “long COVID” syndrome [22]. In our cohort the worsening of exertional dyspnea was similar among the three groups (oxygen only, CPAP and IMV) suggesting that the mechanisms causing breathlessness may be at least partially independent of the severity of pneumonia. These data also suggest the importance of psychological follow-up and rehabilitation programs in patients with a persistent burden of symptoms months after COVID-19 recovery.

In our cohort of patients with COVID-19, we observed a three times higher risk of DLCO impairment in patients with asthma compared to those without this comorbidity. The impact of asthma on COVID-19 remains largely unknown. The available literature suggests that asthma is not associated with worse COVID-19 short-term outcomes, including mortality [23, 24]. However, data on long-term outcomes after COVID-19 in large cohorts of patients with asthma is not yet available. The DLCO impairment we observed in our patients with asthma might be related both to COVID-19 sequelae and asthma itself, although none of the patients presented a disease exacerbation at the time of the follow-up visit. However, in our study, asthmatic patients were only 16 and results should be interpreted with care. When we excluded asthma from the models (due to the low number of asthmatic patients) results were consistent.

Our results and those of the available literature suggest that elderly patients with more severe pneumonia (IMV group) may require a more standardized follow-up including complete PFTs and chest HRCT to better evaluate the presence of long-term pulmonary sequelae. However, even patients with less severe pneumonia (“oxygen only” group) and less functional (DLCO) and radiological involvement, still showed a slight worsening of dyspnea and physical performance at 6MWT between 6- and 12-month follow-up, suggesting that, in case of persistence or appearance of new respiratory symptoms, a personalized follow-up may be required. In this scenario, HRCT scan and DLCO appear as the most sensitive tools to identify pulmonary sequelae.

Among the main strengths of our study we acknowledge (1) the multicentric design, which included both university and non-university hospitals, that increased the generalizability of the results; (2) the selection criteria excluded patients with pre-existing structural lung diseases that may have hampered the possibility of identifying sequelae of SARS-CoV-2 pneumonia.

Our study presents also some limitations: (1) the study visits were conducted during the third pandemic wave and this may have contributed to the lost to follow-up of some patients, however the distribution of age and gender was similar among all patients recruited and patients actually visited; (2) data on the severity of radiological involvement during hospitalization, that may have had an impact on the development of pulmonary sequelae, were not collected; (3) we did not collect any pre-COVID-19 CT scan, therefore it is impossible to evaluate if minor interstitial lung abnormalities, emphysema or bronchiectasis were subclinical pre-existing alterations; (4) We decided to use predefined cut-offs retrieved from the literature to define the presence and degree of PFTs alterations instead of the Global Lung Function Initiative (GLI) guidelines although this choice may have lead to an under- or overestimation of the proportion of patients with impaired PFTs.

In conclusion, we observed in the majority of patients at 12 months from SARS-CoV-2 pneumonia hospitalization minor non-fibrotic chest HRCT abnormalities. DLCO was the most sensitive functional parameter to identify lung sequelae and we observed its improvement between 6- and 12-month follow-up. Nevertheless, a considerable and increasing proportion of patients still reports exertional dyspnea regardless of the initial severity of the disease. Older patients and those who required IMV are at higher risk of developing pulmonary sequelae, however also patients with persistent or worsening respiratory symptoms may require a personalized follow-up. Further studies with longer follow up (2–3 years) are required to evaluate the possible progression of non-fibrotic interstitial lung abnormalities.

## Data Availability

Individual participant data referring to this article (i.e. text, tables and figures) will be made available upon reasonable request. The study protocol will be made available for researchers who provide a methodologically sound proposal. Proposals should be directed to paola.faverio@unimib.it.
